# 2-*O*-Acyl-3-*O*-(1-acyloxyalkyl) Prodrugs of 5,6-Isopropylidene-l-Ascorbic Acid and l-Ascorbic Acid: Antioxidant Activity and Ability to Permeate Silicone Membranes

**DOI:** 10.3390/pharmaceutics8030022

**Published:** 2016-07-18

**Authors:** Nikki A. Thiele, Jennifer McGowan, Kenneth B. Sloan

**Affiliations:** Department of Medicinal Chemistry, University of Florida, P.O. Box 100485, Gainesville, FL 32610, USA; nthiele@ufl.edu (N.A.T.); jmm381@case.edu (J.M.)

**Keywords:** vitamin C, l-ascorbic acid, prodrugs, topical delivery, solubilities, cellular antioxidant activity, cytotoxicity, soft alkyl

## Abstract

2-*O*-Acyl-3-*O*-(1-acyloxyalkyl) prodrug derivatives, **15**, of 5,6-isopropylidene-l-ascorbic acid, **VCA**, and l-ascorbic acid, **VC**, have been characterized by measuring (1) their solubilities in water (S_AQ_) and in 1-octanol (S_OCT_); (2) the ability of one member of the homologous series, **15a**, to diffuse through a silicone membrane from its application in propylene glycol:water (PG:AQ), 30:70; (3) the ability of another member of the series, **15e**, to express cellular antioxidant activity (CAA) in HaCaT cells; and (4) the ability of **15e** to support cell viability in HaCaT cells. All of the prodrugs were more soluble in 1-octanol than **VC** or **VCA** were. **15a**, which exhibited a good balance between S_OCT_ and S_AQ_, was found to deliver approximately 15 times more **15a** than **VCA** delivered **VCA** through a silicone membrane from PG:AQ, 30:70. Under those conditions, no **VC** permeated the membrane. **15e**, which hydrolyzed to release acetaldehyde as a byproduct instead of the toxin formaldehyde, exhibited approximately 30 times the antioxidant activity of **VC** in CaHaT cells and supported cell viability up to 900 μM in HaCaT cells.

## 1. Introduction

**Figure 1:**
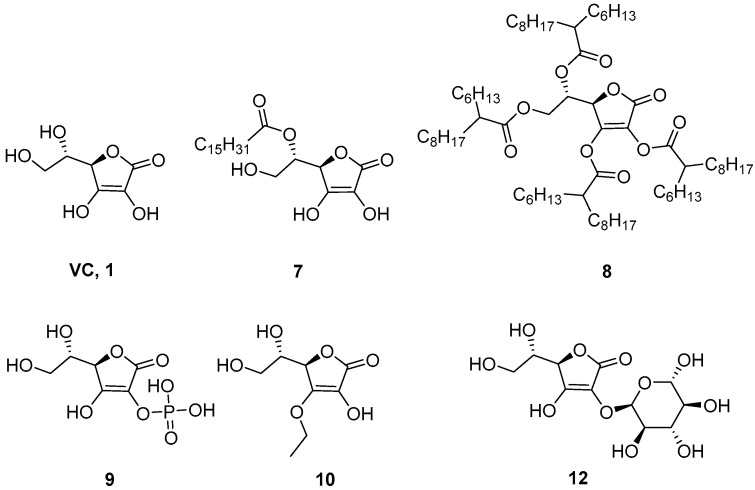
Structures of vitamin C (**VC**, **1**), acylated derivatives (**7**–**9**), and alkylated derivatives (**10**, **12**).

Vitamin C (l-ascorbic acid, **VC**, **1**, Figure 1) is an antioxidant which is frequently used in cosmetic formulations [1]. Not only can **VC** stabilize the formulation against oxidative degradation, but it can also partition into the skin and serve as an antioxidant there. Unfortunately, **VC** is so easily oxidized in cosmetic formulations, especially if the formulation is exposed to air and/or light, that its concentration in those formulations is rapidly depleted, leaving the remaining components unprotected from further oxidation [1]. The depletion of **VC** in the formulation also means that there is little intact **VC** available to partition into the skin and protect the skin from oxidative processes [2]. In addition to its rapid depletion in cosmetic formulations, the physicochemical properties of **VC** are not suitable for achieving sufficient topical delivery for it to be effective. **VC** is a very polar, hydrophilic molecule. It contains a total of four OH groups, two of which are acidic; hence **VC** is significantly ionized at physiological pH. The effect of the four OH groups is that the log partition coefficient (logK_OCT:AQ_) of **VC** is −1.69, where OCT is the lipid 1-octanol and AQ is water [3]. Thus, **VC** is over 50 times more soluble in AQ than OCT under neutral conditions. **VC** is not sufficiently lipophilic (lipid soluble) to effectively partition into the lipophilic barrier to delivery in the skin—the stratum corneum—except under acidic conditions when the OH groups are not ionized and hence **VC** is not as polar [2,4].

**VC**’s antioxidant properties, its instability and its poor physicochemical properties are due to its unique 1-one-2,3-diol-2-ene set of connected functional groups (referred to as the “system” throughout). In the 1-one-2,3-diol-2-ene system the 3-OH group, as a vinylogous carboxylic acid, ionizes first: acidic pKa of 4.17 [5]. Thus at physiological pH, **VC** is 99.9% ionized to its highly polar, water soluble 3-monoanion, **2**. Loss of one electron from **2** gives a radical **3**. Notably, the ionization of the 2-OH group of **3** to form **4** [6] is more facile than is the ionization of the 2-OH group of the 3-monoanion, **2** [5]. This difference is reflected in the acidic pKa values of their 2-OH groups: −0.86 vs. 11.57 for **3** vs. **2**. Finally, loss of another electron from **4** gives the diradical **5**, which rearranges to inactive dehydroascorbic acid **6**. In the process, **VC** gives up two protons and two electrons to quench free radicals being formed in a formulation and/or in the skin.

One way to stabilize the 1-one-2,3-diol-2-ene system in **VC** to premature oxidation and to improve its delivery into the skin is to mask the 3-OH group with chemical derivatives that prevent its ionization at physiological pH to the very polar 3-monoanion **2** [7]. The 2-OH group is not sufficiently acidic (acidic pKa = 11.57) at physiological pH to be significantly ionized as long as the 3-monoanion has not donated an electron to form the radical **3**. However, the 2-OH remains a polar functional group that contributes to the poor lipophilicity of **VC**, so masking it is also desirable. If the masking agents contain lipophilic alkyl groups, these types of masked derivatives of **VC** have the benefit of increasing the lipid solubility (lipophilicity) of the derivative in addition to preventing ionization of the 3-OH group. Furthermore, these masking agents must be transient and revert to **VC** either enzymatically or chemically in a predictable manner that is reasonably rapid and complete. This is because both the 3-OH and the 2-OH groups are essential for the expression of the full complement of the antioxidant properties of **VC**. Such transiently-masked derivatives are called prodrugs and the masking agents are called promoieties.

**Figure 2:**
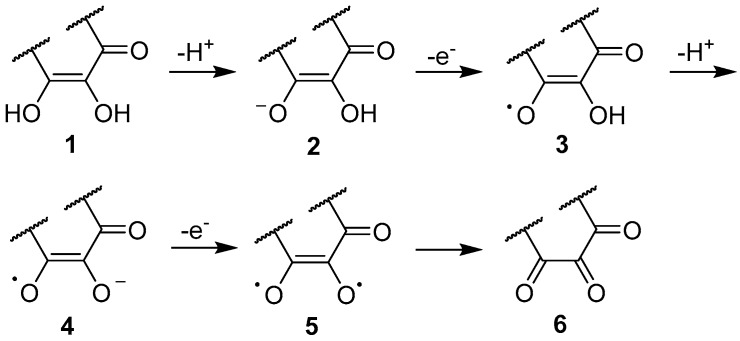
Mechanism for the ionization and oxidation of **VC**, **1**, to inactive dehydroascorbic acid, **6**. Partial structures are shown.

Numerous transient and non-transient derivatives of **VC** have been synthesized through the years to stabilize the 1-one-2,3-diol-2-ene system against oxidation and/or to increase the lipophilicity of **VC** in attempts to make delivery of **VC** into the skin more effective. Alkylcarbonyl derivatives of the 6-OH group in **VC** (such as the 6-*O*-palmitate ester of **VC**, **7**, Figure 1) increase the lipophilicity of **VC** but do not effectively revert to **VC** (when applied topically, they are not prodrugs) and do not effectively stabilize the 1-one-2,3-diol-2-ene system against oxidation. This is because the 3-OH can still ionize and donate electrons as outlined above for **VC** in Figure 2 to give the 6-*O*-palmitate ester of **6** [1]. Alkylcarbonyl and alkyloxycarbonyl derivatives of all four OH groups (such as the 2-*O*-, 3-*O*-, 5-*O*-, 6-*O*- tetra-isopalmitate ester of **VC**, **8**, Figure 1) also increase the lipophilicity and do prevent the ionization of the 3-OH, effectively stabilizing the 1-one-2,3-diol-2-ene system against oxidation. Unfortunately, these tetra acyl derivatives are too large and are too lipid soluble to cross the stratum corneum into the skin: they do not exhibit a good balance between lipid and aqueous solubilities [7]. Also, acylation of the acidic 3-OH group essentially gives a vinylogous carboxylic acid anhydride. Anhydrides are very reactive agents and may cause irritation and potential toxicity if they are applied to the skin. In addition, the 3-*O*-acyl derivatives are likely to be too unstable to formulate, especially for cosmetic application, with the 3-*O*-acyl groups hydrolyzing first as expected for an anhydride versus the 2-*O*-ester. Besides the more lipophilic *O*-acyl derivatives, salts of the acyl-like 2-*O*-phosphate derivative, **9** (Figure 1), have also been marketed. The 2-*O*-phosphate reverts to **VC** by the action of phosphatases. The 2-*O*-phosphate also stabilizes the 3-OH group to ionization, and hence its subsequent rapid oxidation by formation of the radical **3** (Figure 2), not by masking it but by suppressing its ionization by the introduction of the even more acidic P(=O)-OH group (acidic pKa = 2). However, the phosphate derivative is even more hydrophilic than **VC**, so it does not improve the delivery of **VC** across the skin where increased lipophilicity is required [1].

**Figure 3:**
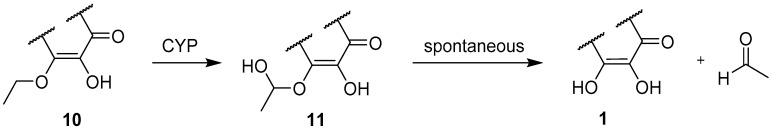
Mechanism for the CYP-mediated reversion of the 3-*O*-ethyl **VC** derivative to **VC**. Partial structures are shown.

A second approach that has been taken to stabilize **VC** to oxidation and to improve its delivery across the skin by making it more lipophilic is to alkylate only the 3-OH or 2-OH group. An example of this approach is the 3-*O*-ethyl **VC** derivative, **10** (Figure 1). The 3-*O*-ethyl derivative masks the 3-OH, preventing its ionization and the subsequent oxidation of **VC**. The 3-*O*-ethyl derivative is also predicted to be more lipophilic than **VC** because of the added lipophilic ethyl group and hence exhibit improved permeation of the skin [1]. However, it is unlikely to deliver **VC** because ethers such as **10** can only undergo reversion to **VC** through a CYP-mediated alpha oxidation of the ethyl group to give an alpha-hydroxyethyl hemiacetal derivative, **11**, which can then spontaneously decompose to an aldehyde (acetaldehyde in this case) and **VC**, **1** (Figure 3). Another alkyl derivative is the 2-*O*-glucoside, **12** (Figure 1). The 2-*O*-glucoside undergoes reversion to **VC** by cellular α-glucosidase. However, the 2-*O*-glucoside is even more polar than **VC** and not more lipophilic because of the addition of four polar OH groups [1]. Thus, it does not improve the delivery of **VC** into the skin. Although the 2-*O*-glucoside does not mask the 3-OH group, it does prevent the second ionization of **VC** (**3** to **4**) as shown in Figure 2, which is a very important step in preventing the oxidation of **VC** to **6**.

Thus, although the importance of stabilizing **VC** against oxidation and of improving its ability to penetrate into skin is well understood by pharmaceutical and cosmetic chemists, to date no chemical derivatives of **VC** have been reported that exhibit all those physiochemical properties (see above) and that also revert to **VC** rapidly and completely. The best approach to stabilizing **VC** against oxidation and improving its delivery into skin that has been reported to date is a formulation approach [2]. The formulation contains **VC**, vitamin E and ferulic acid at a pH of about 3 [8]. At pHs of about 3 the 3-OH group in **VC** is not substantially ionized, so it is not susceptible to the oxidation pathway shown in Figure 2. In addition, if the 3-OH group of **VC** is not ionized, **VC** is a much more lipid soluble (lipophilic) molecule and is thus able to cross the lipophilic barrier to penetration into skin (the stratum corneum) to a much greater extent. At pH 3 **VC** penetrates pig skin in vitro about four times better than it does at pH 5 where **VC** is significantly ionized at the 3-OH group [2]. As attractive as such formulations are, the applications to skin of a neutral derivative of **VC** at pH 7 exhibiting the physicochemical properties listed above, instead of a formulation at pH 3, is very appealing.

Here we report that 3-*O* soft alkyl derivatives of **VC** and 5,6-isopropylidene-l-ascorbic acid, **VCA**, which had been previously synthesized [9,10], exhibit physicochemical and biological properties that suggest that they would be ideal prodrugs of **VC** and **VCA**. The 3-*O* soft alkyl prodrugs mask the 3-OH group to stabilize the 1-one-2,3-diol-2-ene system to oxidation, they increase the lipid solubility of **VC** and **VCA**, and they revert to **VC** and to **VCA** chemically and enzymatically by the action of ubiquitous esterases—unlike the 3-*O*-ethyl derivative (**10**), they are prodrugs. In addition, the 3-*O* soft alkyl prodrug derivatives have been acylated in the 2-*O* position to further enhance the lipid solubilities of the prodrugs and to prevent potential rearrangements of the 3-*O* soft alkyl groups that could be mediated by the 2-OH group. The 2-*O*-acyl groups are also hydrolyzed by esterases.

**Figure 4:**
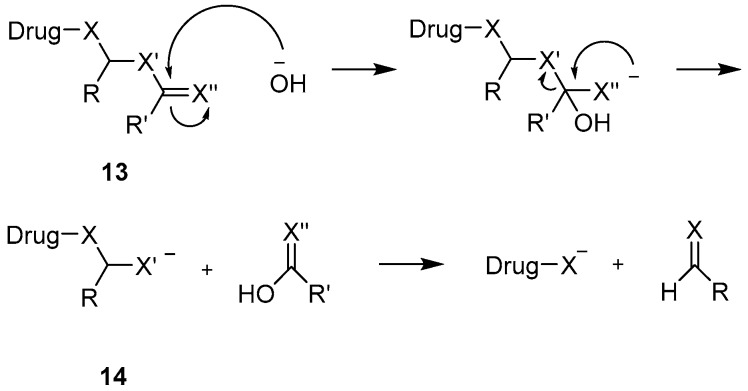
Mechanism for reversion of a soft alkyl derivative of an acidic functional group in a drug molecule.

Soft alkyl types of derivatives have been used for over 35 years to improve the physicochemical properties and the subsequent topical delivery of very polar drug molecules which contain acidic oxygen- or nitrogen-centered functional groups [7]. The design of soft alkyl derivatives takes advantage of the fact that hydrolysis of a functional group in the promoiety attached to a functional group in the parent molecule through a methylene (or vinylogous methylene) group gives an intermediate similar to **11** in Figure 3 which spontaneously reverts to the parent molecule and an aldehyde. A generic example of a soft alkyl derivative of a drug–XH (**13**) is shown in Figure 4 where X, X’ and X” can be O, N or S, and R and R’ can be hydrogen, alkyl or aryl groups. In addition, R’ can be alkyloxy groups. Attack by a hydroxide anion or an esterase on the polarized C(=X”) functional group leads to a cascade of rearrangements which ultimately result in the release of drug-XH, RCH(=X’) and R’C(=X”)OH. Most often, X’ and X” are oxygen so that the byproducts are an aldehyde and a carboxylic acid. Protonation of the intermediate **14** gives the same type of intermediate as **11** in Figure 3. Thus, soft alkyl prodrugs are stabilized forms of a hemiacetal such as **11**.

**Figure 5:**
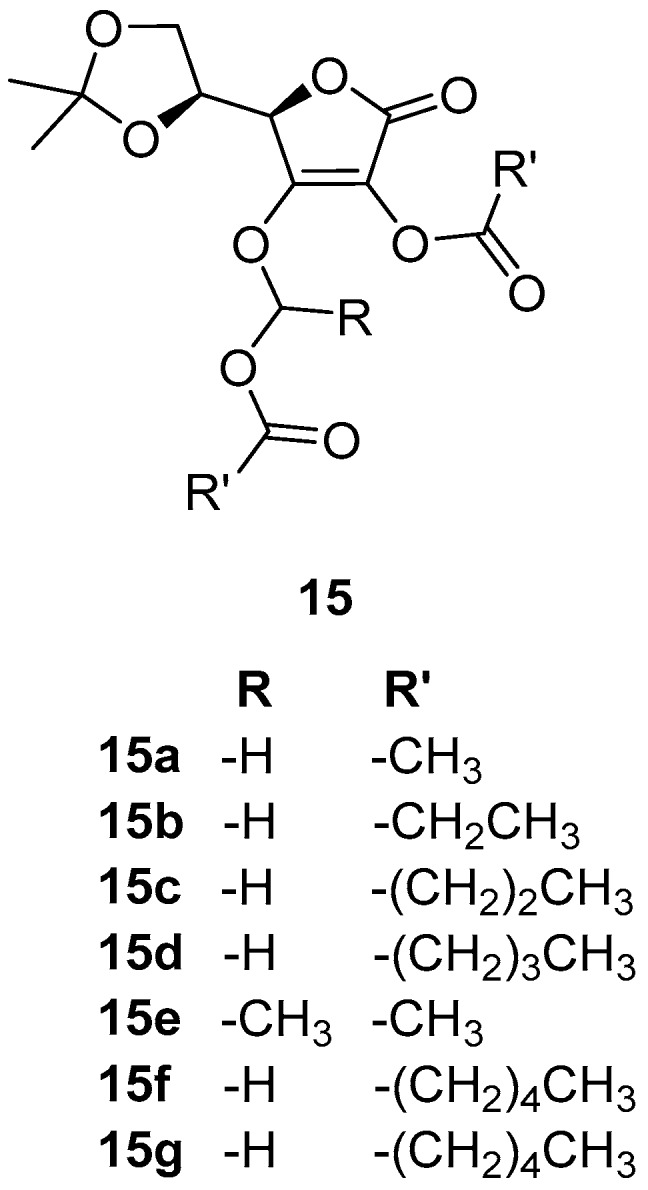
2-*O*-acyl-3-*O*-(1-acyloxyalkyl) derivatives of VCA, **15a**–**g**.

The specific 3-*O*-soft alkyl derivatives, which are characterized here, are the 2-*O*-acyl-3-*O*-(1-acyloxyalkyl) derivatives of **VCA**, **15** (Figure 5) [9,10]. The regioselective preparation of **15** have been previously reported [10]. Here we report some preliminary results for (1) the water, S_AQ_, and the 1-octanol, S_OCT_, solubilities of a homologous series of **15**, (2) the ability of a representative member of the homologous series of **15** (2-*O*-acetyl-3-*O*-acetyloxymethyl, **15a**) to permeate a silicone membrane from its application in propylene glycol:water (PG:AQ), 30:70 [11,12], and (3) the ability of a different representative member of the homologous series of **15** [2-*O*-acetyl-3-*O*-(1-acetyloxyethyl), **15e**] to inhibit oxidation in the cellular antioxidant assay (**CAA**) while maintaining low cytoxicity in the cell viability assay (**CVA**).

## 2. Experimental

Reagents and solvents used in this report were purchased from Sigma-Aldrich (St Louis, MO, USA), were of reagent grade and used without further purification. The Franz diffusion cells (surface area 4.9 cm^2^, receptor volume 20 mL) were obtained from Crown Glass (Somerville, NJ, USA). Silicone membranes (0.254 mm) were obtained from Pillar Surgical (La Jolla, CA, USA). The solvents and the water bath used with the diffusion cells were obtained from Fisher Scientific (Pittsburg, PA, USA). The ^1^H and ^13^C-NMR spectra were run on a Varian Mercury, 400 MHz, spectrometer. The ultraviolet (UV) spectra were run on a Shimadzu UV-2550 spectrophotometer. **VCA** was also purchased from Sigma-Aldrich or synthesized by a previously published method [13]. The synthesized **VCA** was identical with the purchased **VCA** by TLC, mp and ^1^H-NMR.

## 3. Methods

### 3.1. Synthesis

*Cleavage of the 5,6-isopropylidene group in 2-O-hexanoyl-3-O-hexanoyloxymethyl-5,6-isopropylidene-l-ascorbic acid, **15f**.* **15f** was synthesized according to a previously published procedure [9]. The 5,6-isopropylidene group was cleaved according to a previously published procedure [14]. To an acetonitrile (10 mL) solution containing 14 mg (0.06 mmole) of antimony trichloride was added **15f** (275 mg, 0.6 mmole) followed by water (10 μL, 0.56 mmole). The reaction mixture was stirred at room temperature for 6 h, quenched with 0.5 mL of saturated sodium bicarbonate and diluted with 50 mL of dichloromethane. The precipitate was removed by filtration and the filtrate was concentrated *in vacuo* without heating to give 180 mg of the desired 2-*O*-hexanoyl-3-*O*-hexanoyloxymethyl-l-ascorbic acid as an oil in 75% yield, which was one component by TLC (R_f_ 0.48, ethyl acetate:hexanes, 1:1) and which gave the correct elemental analysis. ^1^H-NMR (CDCl_3_) δ 5.8 (2*H*, dd, –O–CH_2_–O–), 4.91 (1*H*, d, 4-CH), 4.0 (1*H*, dt), 3.85 (1*H*, dd), 3.80 (1*H*, dd), 2.55 (2*H*, t, CH_2_–C(=O) –O), 2.35 (2*H*, t, CH_2_–C(=O)–O).

### 3.2. Solubilities

Direct solubilities were measured according to previously published procedures [15]. Briefly, excess prodrugs **15a**–**d**, **VC**, **VCA** or **VC** 6-palmitate were suspended in 1-octanol, OCT, or water, AQ, and the suspensions were stirred for 1 h in the case of the prodrugs in AQ or for 24 h in the case of the prodrugs in OCT. Excess **VC**, **VCA** or **VC** 6-palmitate were suspended in OCT or water and stirred for 24 h before the suspensions were filtered. Excess **VC**, **VCA** and prodrug **15a** were also suspended in propylene glycol (PG):water (AQ), 30:70, and stirred for 24 h before the suspensions were filtered. All of the suspensions were stirred at 23 ± 1 °C. All of the saturated solutions were diluted with AQ or acetonitrile (ACN) and the UV absorption of each solution was measured. The saturated concentration of each compound was determined using Beer’s Law and the previously measured molar absorptivities (ε, L·mol^−1^ or M^−1^) of each compound in AQ or ACN to give S_OCT_, S_AQ_ or S_30:70_.

Indirect solubilities were also estimated by first measuring the partition coefficients of the molecules between OCT and AQ according to previously published procedures [15]. Briefly, the UV absorptions of the saturated solutions of the compounds, calculated from the UV absorptions of the samples diluted with AQ or ACN to keep absorption values between 0.2 and 2, in either OCT or AQ were taken. Then, the saturated solutions were partitioned against the other solvent (OCT against AQ, or AQ against OCT) and the UV absorptions of the initial saturated solutions (OCT or AQ, respectively) were measured again after separation of the two solvents and dilution of the OCT or AQ phases, respectively. The resulting partition coefficients were calculated as absorbances before (A_B_) and absorbances after (A_A_) multiplied by the inverse of the ratios of the volumes of the two solvents used (V_OCT_ or V_AQ_). Thus, if OCT was the initial saturated solution:

K_OCT:AQ_ = A_A_/(A_B_ − A_A_) × (V_AQ_/V_OCT_)

When the initial saturated solution was AQ, K_AQ:OCT_ was obtained, from which K_OCT:AQ_ could be calculated by taking the inverse of K_AQ:OCT_. Assuming that the solubility ratio (SR) and the partition coefficient are essentially equivalent (SR_OCT:AQ_ = K_OCT:AQ_), then estimated S_AQ_ = S_OCT_/K_OCT:AQ_ or S_OCT_ = (K_OCT:AQ_)(S_AQ_).

### 3.3. Diffusion Cell Experiments

The diffusion cell experiments were run according to a previously described procedure using Franz diffusion cells maintained at 32 °C with a circulating water bath, silicone as the membrane and PG:AQ, 30:70, as the donor and the receptor phases [16]. Briefly, the donor suspensions were prepared by suspending 1.2 g of **VC**, 250 mg of **VCA** or 60 mg of **15a** in 4 mL of PG:AQ, 30:70, for either 24 h for **VC** or 2 h for **VCA** or **15a** with stirring at 23 ± 1 °C. An aliquot (1.0 mL) of each suspension was applied to the donor side of each of three diffusion cells (*n* = 3) and the donor phases were sealed with Parafilm. The donor phases remained as suspensions throughout the experiments. Samples were taken from the receptor phases every 10–12 h after application and the receptor phases were changed after each sampling. After sample acquisition, the donor phases of **VC**, **VCA** and **15a** were always replaced with freshly prepared donor suspensions. After 4–5 samples were taken, the donor phases were removed, the membrane surfaces were washed with CH_3_OH and the membranes were leached with CH_3_OH for 72 h to remove residual **VC**, **VCA** or **15a** (or **VCA** from the hydrolysis of **15a**) from the silicone membranes.

A second application (1.0 mL) of a suspension of 400 mg of theophylline in 6 mL of PG was made to each membrane after the leaching period was complete. The second applications part of the experiment were run to determine if any damage to the integrity of the membranes had been caused by the first applications [17]. Samples of the receptor phases were taken every 24 h after the second application until 3 samples had been acquired. The receptor phases were changed after each sample acquisition.

The amounts of **VC**, **VCA** or **15a** in the diffusion cells at each sampling time for the first applications and the amounts of theophylline for the second applications were determined from their UV absorption at their respective λ_max_ in PG:AQ, 30:70, using the molar extinction coefficients that had been previously measured. Flux values were calculated from the slopes of the plots of cumulative amounts versus time divided by the surface area of the membranes to give the maximum flux through a silicone membrane from PG:AQ, 30:70: J_MP 30:70_ in μmole·cm^−2^·h^−1^. Steady state was taken as from 11 to 45 h for the first applications and from 24 to 72 h for the second applications. The second application fluxes were found to be within the standard deviation reported for the literature value for the maximum flux of theophylline through a silicone membrane from its suspension in propylene glycol (PG): log J_MPPG_ = −2.68 +/− 0.12 μmole·cm^−2^·h^−1^ [15]. No values for log J_MPPG_ different from −2.68 ± 0.12 μmole·cm^−2^·h^−1^ were observed.

### 3.4. Cellular Antioxidant Activity (CAA) Assay and Cell Viability Analysis (CVA) Experiments

The CAA assay and CVA experiments were each performed by Brunswick Labs (Southbough, MA, USA) under the supervision of Dr. Jin Ji using previously reported procedures [18,19].

Briefly, the CAA assay was run using HaCaT cells instead of HepG2 cells as previously reported [20]. Triplicate wells containing the HaCaT cells were treated for 24 h with 100 μL of **15e** or quercetin in 16:84, PG:HBSS (Hanks’ Balanced Salt Solution) plus 25 μM DCFH-DA (2’,7’-dichlorofluorocin diacetate) in HBSS. Then 600 μM ABAP (2,2’-azobis (2-amidinopropane) dihydrochloride) in 100 μL of HBSS was added and the 96-well microplate was placed into a Fluoroskan Ascent FL plate-reader (ThermoLabsystems, Franklin, MA, USA) at 37 °C. Emission at 538 nm was measured with excitation at 485 nm every 5 min for 1 h. Control wells contained cells treated with DCFH-DA and ABAP. After blank subtraction from the fluorescence readings, the area under the curve of fluorescence versus time was integrated to calculate the CAA value at each concentration as follows:

CAA unit = 100 − (∫ SA/∫ CA) × 100

where ∫ SA is the integrated area under the sample fluorescence versus time curve and ∫ CA is the integrated area under the control fluorescence versus time curve. The median effective dose (EC_50_) was determined from the plot of log (f_a_/f_u_) versus log dose, where f_a_ is the fraction affected and f_u_ is the fraction unaffected by the sample treatment at each concentration of **15e** or quercetin. In each experiment quercetin was used as the standard and cellular antioxidant activity for **15e** was expressed as micromoles of quercetin equivalents (QE) per 100 micromoles of **15e**.

Briefly, in the CVA analysis HaCaT cells were treated with a series of concentrations of **15e** for 24 h [19]. The degree of cell viability was assessed by measuring the amount of cellular adenosine triphosphate (ATP) remaining after treatment with **15e**, where ATP functions as a biomarker for metabolically active cells. The maximum concentration of **15e** used to treat the cells that decreased the ATP bioluminescence absorbance by less than 10% was considered to be non-cytotoxic at that concentration.

## 4. Results and Discussion

### 4.1. Synthesis

**Scheme 1:**
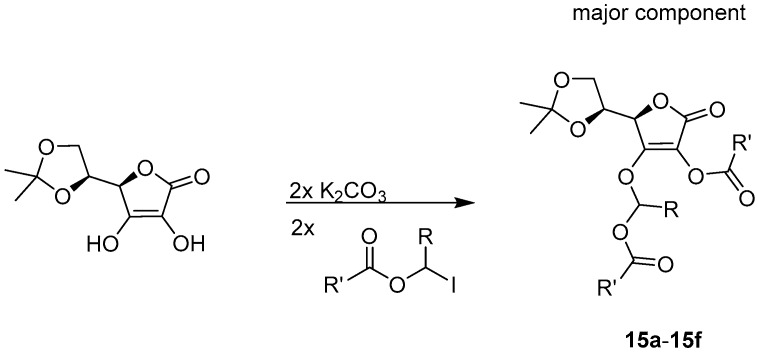
Pathway A for the synthesis of 2-acyl-O-(1-acyloxyalkyl)-5,6-l-ascorbic acid derivatives, **15**.

**Scheme 2:**
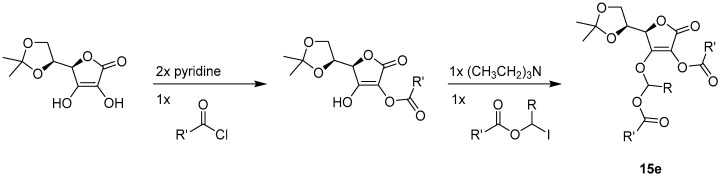
Pathway B for the synthesis of 2-acyl-O-(1-acyloxyalkyl)-5,6-l-ascorbic acid derivatives, **15**.

The various 2-acyl-3-*O*-(1-acyloxyalkyl)-5, 6-l-ascorbic acid derivatives, **15**, used in these experiments were synthesized by either Pathway A [9] or Pathway B [10], shown in Scheme 1 and Scheme 2, respectively.

The prodrugs **15a**–**e** that are characterized here were all synthesized from the reaction of two equivalents of (1-acyloxyalkyl)-1-iodide with one equivalent of **VCA** in acetone using K_2_CO_3_ as base in a heterogeneous reaction (Scheme 1). These reactions gave a complex mixture of products from which the major components, **15a**–**e**, were isolated, frequently in only low yield. Subsequently, a straight forward, two-step synthesis was developed that cleanly gave much better yields of **15** in which R’ at the 2-*O* and 3-*O* positions can be varied independently to give more diverse structures for **15** (Scheme 2). However, **15a**-**d** were not resynthesized for use in the determination of their solubilities (Section 4.2) using the improved two-step process.

The cleavage of the 5,6-isopropylidene group from **15** to give a prodrug of **VC**, instead of **VCA**, had not been previously reported. Here we report that the 5,6-isopropylidene group of 2-*O*-hexanoyl-3-*O*-hexanoyloxymethyl-5,6-isopropylidene-l-ascorbic acid can be easily removed using a catalytic amount of antimony trichloride and one equivalent of water. The isolation of the product in good yield required only quenching the reaction with bicarbonate, filtering the resulting reaction mixture and concentrating the filtrate. Thus, the 2-*O*-acyl-3-*O*-(1-acyloxyalkyl) derivatives of l-ascorbic acid can be easily obtained and can serve as prodrugs of l-ascorbic acid, **VC**, itself in addition to the 2-*O*-acyl-3-*O*-(1-acyloxyalkyl)-5,6-isopropylidene derivatives of l-ascorbic acid serving as prodrugs of **VCA**. In either case the prodrugs protect the 1-one-2,3-diol-2-ene system from premature oxidation and impart increased lipophilicity to either **VC** or **VCA** to improve their delivery. Importantly, either **VC** or **VCA** express the full complement of the antioxidant properties of the 1-one-2,3-diol-2-ene system in **VC**.

### 4.2. Solubilities

The solubilities in 1-octanol and water (S_OCT_ and S_AQ_, respectively) and the log partition coefficients between OCT and AQ (log K_OCT:AQ_) that were determined for **15a**–**e** (Table 1) show that the data for the series is consistent from one member of the series to the next. In homologous series of prodrugs such as **15a**–**d**, as CH_2_ groups are sequentially added to n-alkyl groups in the promoieties, the difference between the log K_OCT:AQ_ of the first member of the series and the next member of the series remains a constant-methylene π [15]. The value of methylene π for log K_OCT:AQ_ measurements is about 0.55 log units. For the present homologous series of prodrugs **15a**–**d**, the average of the difference between contiguous members of the series was 1.16 +/− 0.04 log units. Since two CH_2_ groups were added to the prodrugs as R’ was increased by one CH_2_ group, the methylene π value for the **15a**–**d** series is half of 1.16 log units: 0.58 log units. This value is consistent with methylene π values for other series of prodrugs [15]. Also as expected, as CH_2_ groups were added the S_OCT_ and the log K_OCT:AQ_ values increased and the S_AQ_ values decreased [15].

For **15e**, the S_AQ_ value was measured directly. The log K_OCT:AQ_ value for **15e** of 0.57 was estimated from the log K_OCT:AQ_ value of −0.01 for **15a**, which had been measured directly, and the methylene π value of 0.58 log units which has been calculated from the log K_OCT:AQ_ values for the **15a** to **15d** series. Then the S_OCT_ value for **15e** was calculated from (K_OCT:AQ_)(S_AQ_) to give 60.6 mM. Both **15a** and **15e** exhibited a good balance of lipid and aqueous solubilities (S_OCT_/S_AQ_ = 0.98 and 3.72, respectively) as well as being the two smaller molecules in the series [7]. Thus, they are both good candidates to increase the delivery of **VCA**.

### 4.3. Diffusion Cell Results

The diffusion cells were run in triplicate using propylene glycol:water (PG:AQ), 30:70, as the donor and receptor phases [11,12]. Silicone membranes were used as a surrogate for human skin. Because of its good balance between S_OCT_ and S_AQ_, **15a** was chosen to represent the series of prodrugs to determine the effectiveness of this type of prodrug. Suspensions of each compound (**VC**, **VCA** or **15a**) were applied to maximize the thermodynamic activity of each compound in the vehicle and in the membrane.

As expected (Table 2), we found that no discernable **VC** was observed in the receptor phases of the cells to which **VC** had been applied. Also as expected, since **VCA** was about 4 times more soluble in the lipid 1-octanol than **VC**, a moderate flux of **VCA** was observed under the same conditions. Finally, the delivery of intact **15a** was about 15 times more effective than the delivery of **VCA** by **VCA**. Thus, the delivery of molecules containing the transiently masked 1-one-2,3-diol-2-ene system is much more effective than the delivery of molecules in which that very polar system is not masked.

### 4.4. Cellular Antioxidant Activity (CAA) and Cell Viability Analysis (CVA)

Although there are a wide variety of chemical antioxidant activity assays, many are not performed at physiological pH and none of them are capable of taking into account bioavailability, cellular uptake and metabolism of the antioxidant. In the case of prodrugs that are meant to function inside cells after they have been metabolized by hydrolysis, an assay that also measures activity based on the ability of the test molecule to cross a lipid biological membrane is essential. In the **CAA** assay using HepG2 cells [18], **VC** exhibits an EC_50_ value (67.5 μM) that is about 10 times greater (10 times less potent) than the standard, quercetin. Usually, the results from the **CAA** assay are converted to μmole quercetin equivalents/100 μmol of the tested antioxidant (QE) where the value for quercetin is set at 100 QE. In terms of QE values, **VC** only gave a value of 4 QE, making it 25 times less potent than quercetin by that criteria. In the **CAA** assay performed here using the HaCaT cell line (a spontaneous transformed aneuploid immortal keratinocyte cell line), which would be more appropriate for measuring topical antioxidant activity, **VC** only gave a value of about 2 QE [20].

In order to measure the **CAA** potency of the soft alkyl type of prodrugs, **15e** was used as the representative of the series. The hydrolysis of **15e** produces acetaldehyde, which is much less toxic than the formaldehyde produced from the hydrolysis of **15a**. When **15e** was tested in the **CAA** assay using HaCaT cells, the QE value was 62 μmole equivalents of quercetin/100 μmole of **15e**. Thus, **15e** was about 30 times more potent than **VC** in the **CAA** assay, which requires the tested molecule to cross a biological membrane to express its activity. In this case, it also means that the prodrug hydrolyzed to the active **VCA** once it crossed the membrane into the cell: **15e** was functioning as a prodrug.

The normal plasma concentration of **VC** is about 50 μM. When **15e** was tested in the **CVA** assay using HaCaT cells, the maximum concentration of **15e** that maintained or promoted cell viability was 920 μM. Thus, no toxicity for **15e** was observed at a concentration that was 20 times greater than the normal concentration of **VC** in plasma.

## 5. Conclusions

A true prodrug of the highly polar 1-one-2,3-diol-2-ene set of connected functional groups in 5,6-isopropylidene-l-ascorbic acid (**VCA**) and l-ascorbic acid (**VC**) has been identified. 2-*O*-Acyl-3-*O*-(1-acyloxyalkyl) derivatives of **VCA** (**15a**–**e**) and **VC** (**15g**) have been regioselectively synthesized. The water and lipid solubilities (S_AQ_ and S_OCT_) have been measured and several molecules in the series of prodrugs that exhibit the balanced lipid and aqueous solubilites necessary for improved delivery across biological membranes [7] have been identified. One representative of the series (**15a**) has been shown to enhance the delivery of **15a** across a silicone membrane from propylene glycol:water, 30:70 by about 15 times compared to the delivery of **VCA** by **VCA** across the same membrane from the same vehicle. Another representative of the series (**15e**) has been shown to inhibit cellular oxidation about 30 times better than **VC** while at the same time maintaining low toxicity at a concentration 20 times greater than the normal concentration of **VC** in plasma. The soft alkyl type of prodrug of **VC** and its derivatives effectively cross HaCaT membranes and decrease cellular oxidation without causing decreased cell viability. They fill the need for molecules with these properties that had not been met before.

## Figures and Tables

**Table 1:** Molar extinction coefficients in water (ε_AQ_) or in acetonitrile (ε_ACN_), solubilities in water (S_AQ_) and in 1-octanol (S_OCT_) and log partition coefficients between 1-octanol and water (logK_OCT:AQ_) for l-ascorbic acid derivatives.

Compound	R	R’	λ_max_ (ε_AQ_) ^a,b^	λ_max_ (ε_ACN_) ^a,b^	S_OCT_^c^	S_AQ_^c^	log K_OCT:AQ_
**15a**	–H	–CH_3_	224 (10,775)	217 (11,012)	35.8 ^d^	36.5 ^e^	−0.01 ^e^
**15b**	–H	–CH_2_CH_3_	224 (10,367)	220 (10,475)	87.5 ^d^	6.46 ^e^	1.13 ^e^
**15c**	–H	–(CH_2_)_2_CH_3_	224 (11,712)	220 (12,327)	437 ^d^	2.04 ^e^	2.33 ^e^
**15d**	–H	–(CH_2_)_3_CH_3_	224 (10,775)	220 (11,060)	2387 ^e^	0.94 ^f^	3.40 ^e^
**15e**	–CH_3_	–CH_3_	225 (10,389)	221 (10,425)	60.6 ^g^	16.3 ^e^	0.57 ^h^
**15f** ^i^	–H	–(CH_2_)_4_CH_3_	ND	ND	ND	ND	ND
**15g** ^i,j^	–H	–(CH_2_)_4_CH_3_	ND	ND	ND	ND	ND
**VC** 6-palmitate			244 (9012) ^k^	238 (9374)	57.8 ^e^	0.008 ^f^	3.85 ^e^

^a^ Wavelength in nm; ^b^ Molar extinction coefficients in L·mole^−1^ or M^−1^; ^c^ Concentrations in mM; ^d^ Calculated from the product of the measured partition coefficient between 1-octanol and water (K_OCT:AQ_) and the measured S_AQ_; ^e^ Measured directly; ^f^ Calculated from the measured S_OCT_ divided by the measured K_OCT:AQ_; ^g^ Calculated from the product of the estimated K_OCT:AQ_ and the measured S_AQ_; ^h^ Estimated from the average of the contribution of each CH_2_ group to the difference between logK for contiguous members of a homologous series; ^i^ λ_max_ (ε_AQ_), λ_max_ (ε_ACN_), S_OCT_, S_AQ_ and log K_OCT:AQ_ were not measured; ^j^ **15g** is **15f** with the acetonide removed; ^k^ Molar extinction coefficient in methanol.

**Table 2:** Molar extinction coefficients in propylene glycol:water (PG:AQ), 30:70 (ε_30:70_), solubilities in 1-octanol (S_OCT_) and in water (S_AQ_) and maximum flux values through silicone membranes from PG:AQ, 30:70 (J_MP 30:70_) of **VC**, **VCA** and **15a**.

Compound	MW	λ_max_ (ε_30:70_) ^a,b^	S_OCT_^c^	S_AQ_^c^	J_MP 30:70_^d^
**VC**	176	258 (8,855)	4.93	1659	- ^e^
**VCA**	216	266 (11,823)	19.7	228	0.0019
**15a**	330	224 (11,473)	35.8	36.5	0.028

^a^ Wavelength in nm; ^b^ Molar extinction coefficients in PG:AQ, 30:70, in: L·mole^−1^ or M^−1^; ^c^ Concentration in mM; ^d^ Flux values in μmole·cm^−2^·h^−1^; ^e^ No flux observed.
